# A Review of Biotechnological Applications of *Glomeromycota*: Inoculum Production, Plant Performance, Environmental Management, and Metabolites

**DOI:** 10.3390/jof12100777

**Published:** 2026-10-16

**Authors:** Cícero Pinheiro Inácio, Greicilene Maria Rodrigues Albuquerque, Maria Alice Ribeiro Alves, Isabelly Guimarães Silva, Geuda Barbosa da Silva, Delson Laranjeira, Rejane Pereira Neves

**Affiliations:** 1 Department of Mycology, Federal University of Pernambuco, Av. Prof. Moraes Rego, s/n, Cidade Universitária, Recife 50670-901, PE, Brazil; greicilene.albuquerque@ufpe.br (G.M.R.A.); mariaalice.alves@ufpe.br (M.A.R.A.); isabelly.gsilva@ufpe.br (I.G.S.); geuda.barbosa@ufpe.br (G.B.d.S.); 2 Department of Phytopathology, Federal Rural University of Pernambuco, Rua Dom Manuel de Medeiros, s/n, Dois Irmãos, Recife 52171-900, PE, Brazil; delson.laranjeira@ufrpe.br

**Keywords:** *Glomeromycota*, arbuscular mycorrhizal fungi, inoculum quality, plant genotype, transcriptomics, phytomanagement, plant metabolites

## Abstract

Arbuscular mycorrhizal fungi (*Glomeromycota*) offer routes to inoculum production, crop management, phytomanagement of contaminated soils, and modulation of plant metabolites. This critical narrative review integrates 102 selected original studies, including genomic, physiological, transcriptomic, cultivation, and field research. Evidence is interpreted by fungal strain, host genotype, tissue, environment, and application-specific endpoint. Host-lipid transfer and asymbiotic propagation inform inoculum manufacture, but spore production remains distinct from infective potency, storage stability, and economic feasibility. Receptor signaling and functional nutrient-transport studies connect symbiotic establishment to resource exchange; stress experiments reveal context-dependent changes in water transport, ion homeostasis, photosynthesis, and oxidative regulation. Commercial-product variability, resident microbiota, pesticide exposure, and ecological responses constrain translation. Metal localization and transport studies distinguish retention, mobilization, and tolerance from contaminant removal. Plant-metabolite experiments demonstrate chemical modulation rather than direct fungal biosynthesis or therapeutic efficacy. Soil carbon and hydraulic responses further show that ecological benefits cannot be generalized across communities or substrates. Development should prioritize verified inocula, defined host × fungal strain × environment combinations, relevant controls, and repeatable field outcomes. The narrative design and author-proposed assessment framework covers a deployment protocol.

## 1. Introduction

Arbuscular mycorrhizas are associations between plant roots and fungi assigned to *Glomeromycota*. The name *Glomeromycotina* also appears in classifications of these lineages, reflecting differences in taxonomic rank and usage. For a review focused on their biotechnology, taxonomic precision matters: “mycorrhizal fungi” also encompasses organisms that form ectomycorrhizal, ericoid, and orchid-associated symbioses. Studies should be assigned to the present scope on the basis of fungal identification and type of association, rather than the general term “mycorrhiza”. Genomic comparisons have clarified the evolutionary history of arbuscular mycorrhizal fungi and characteristics associated with their symbiotic lifestyle [1].

Applications extend from inoculum production to crop management, phytomanagement of contaminated sites, and modulation of plant secondary metabolism. Each use calls for an endpoint matched to its purpose. Crop trials require harvest-relevant performance; remediation studies need measurements of contaminant fate and exposure; and chemical-production studies must examine verified total yield and consistency. Improved root colonization or early biomass can support an explanation for an effect, but neither necessarily establishes a useful final outcome.

Environmental context is a further obstacle to translating experimental success into routine use. Across 54 maize fields, responses to arbuscular mycorrhizal inoculation varied substantially, and indicators of the resident soil microbiome helped account for part of that variation [2]. Performance with one fungal isolate, host genotype, or soil cannot simply be extended to another. Nutrient management, resident microorganisms, and local growing conditions must inform both candidate selection and experimental controls.

This relationship offers several routes to application. Arbuscular mycorrhizal fungi can influence the acquisition of soil-derived nutrients and alter plant growth or responses to stress, while the host supplies resources required by the fungus. Experimental work on plant-derived fatty acids and lipid biosynthesis has made this exchange particularly relevant to inoculum production [3,4]. Yet a demonstrated mechanism does not establish that an inoculant will benefit every crop. The outcome also depends on whether viable propagules establish in the intended host and function under the conditions in which the plant is grown.

This review critically examines original studies involving *Glomeromycota* across inoculum production, crop cultivation, phytomanagement of contaminated soils, and modulation of plant metabolites for biotechnological applications. It distinguishes evidence explaining how an association may function from evidence showing that an intervention produces a useful outcome. The central questions are which effects have been measured, under what conditions they change, and what remains to be established before an inoculation practice can be developed for wider use. Studies exclusively involving other mycorrhizal groups fall outside this scope.

## 2. Review Scope and Approach

### 2.1. Literature Identification and Review Design

This article is a critical narrative review of selected original research, not a systematic review, scoping review, or meta-analysis. The initial literature-identification exercise used PubMed searches recorded on 23 September 2026 for publications from 23 September 2016 onward, together with directed SciELO searches. During revision, targeted DOI-based checking of publisher articles, PubMed records, and Crossref metadata added eight studies. A further thematic extension on 2 October 2026 used Europe PMC searches of MEDLINE-indexed records and targeted publisher/PubMed checking for original research published from 23 September 2016 to 2 October 2026, of Web of Science, Scopus, or CAB Abstracts. Themes included lipid metabolism and cultivation, genomics, signaling and nutrient transport, crop management, abiotic and biotic stress, contaminants, plant metabolites, and soil ecological outcomes.

### 2.2. Selection Principles and Limits of Coverage

The selected evidence set comprises 102 original articles: the 28 studies in the first revision and 74 additional studies chosen for their contribution to the mechanistic or application-focused discussion. Selection was purposive and was not intended to estimate the frequency of beneficial outcomes. Original empirical, observational, and comparative genomic research was eligible when it investigated an arbuscular fungus, an explicitly described arbuscular inoculum, or a directly relevant arbuscular-symbiosis mechanism. Broader plant-lineage comparisons were retained only for their arbuscular-signaling evidence. Searches excluded records indexed as reviews and titles identifying reviews or meta-analyses; article type and relevance were then checked against indexed abstracts and bibliographic records. Citation-ranked retrieval helped locate established mechanistic studies and was not treated as an unbiased sampling procedure.

Reviews, meta-analyses, theses, conference abstracts, and studies exclusively involving ectomycorrhizal, ericoid, or orchid-associated fungi were excluded from the empirical synthesis. Bacterial co-inoculation alone was not grounds for exclusion: a study was outside the attribution scope when an arbuscular component was absent or its contribution could not be separated from the other intervention. A taxonomically uncertain report was not treated as species-specific evidence unless the source explicitly identified an arbuscular fungus or association. Broad retrieval totals and automated relevance flags from the search are not presented as a PRISMA flow.

DOIs, titles, authors, journal details, and publication dates were checked against Crossref metadata and indexed or publisher records. Issue publication years were used, with online dates retained for eligibility assessment. Scientific statements were limited to the source material inspected; indexed abstracts were used where full text was not available, and no unreported dose, identification assay, or control was inferred. Table 1 provides a detailed map of 28 illustrative studies retained from the first revision, while the additional evidence is integrated into the narrative and complete reference list. M, C, and F describe mechanistic, controlled, and field settings for that map; they are not formal quality grades. No independent duplicate screening, pooled effect estimate, or global evidence-certainty score was performed. Selective retrieval, access limitations, differing designs, and possible overlap among related experiments constrain the synthesis.

## 3. Critical Synthesis

### 3.1. Scope of the Evidence Discussed

The 102 selected original studies address fungal propagation, genomics, nutrient exchange, product quality, crop performance, stress-related molecular responses, contaminated-soil management, plant metabolites, and ecological outcomes. The larger evidence base permits comparison of complementary mechanisms and contrasting responses, but does not represent all research in these areas. Positive, absent, and unfavorable responses are interpreted within their experimental contexts rather than pooled into a single estimate of efficacy.

Table 1 details 28 illustrative studies spanning the application domains and recent transcriptomic findings; it is not a row-by-row inventory of all 102 references (Table 1). The additional studies are discussed in the relevant subsections. Metabolite identification and plant gene-expression measurements are distinct from fungal taxonomic identification and are not presented.

**Figure 1:**
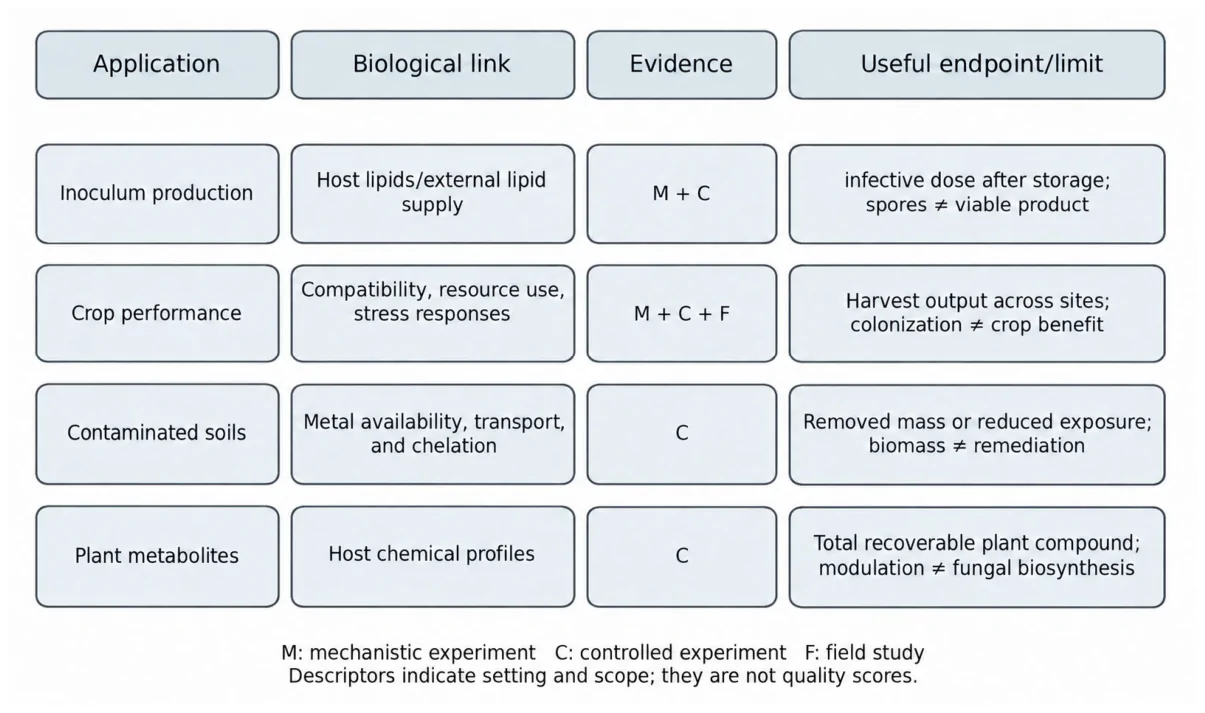
Application–mechanism–endpoint map constructed from the selected original studies. Supporting studies address inoculum production [3,4,5,21], crop performance [2,7,8,9,10,11,12,23,24,25,27], contaminated soils [13,14,15], and plant metabolites [16,17,18,19,20]. M denotes mechanistic evidence, C controlled proof of concept, and F field observations; these categories describe study setting and scope, not a formal risk-of-bias grade. The figure does not pool effect sizes or imply equivalent support across domains.

The detailed map separates named inocula, experimental verification, and measured utility. Molecular tracking strengthens attribution to an introduced isolate, whereas a fungal name alone does not establish an identification method. Studies measure different endpoints—infective propagules, nutrient flux, host responses, metal behavior, plant compounds, or product performance—and these cannot be collapsed into one measure of biotechnological success. The expanded narrative compares these endpoints across complementary genomic, physiological, and ecological experiments (Figure 1).

### 3.2. Production and Tracking of Arbuscular Mycorrhizal Inoculum

Producing an effective inoculum requires more than generating fungal biomass or spores. The material must contain viable propagules, retain infectivity after formulation and storage, and establish in the intended host. This is particularly relevant to arbuscular mycorrhizal fungi because their development is closely linked to plant-derived resources. Experimental evidence of host-to-fungus fatty-acid transfer and work implicating rice OsRAM2 in lipid biosynthesis and symbiotic development clarify part of this dependency [3,4]. These findings can guide propagation research, but they do not show that every isolate responds identically to a cultivation substrate.

The mechanisms also clarify why a gain in fungal biomass outside the root cannot be taken as proof of a functional product. Infective capacity must be measured after propagation and again after formulation or storage, under conditions representative of the intended application.

Asymbiotic mass production of *Rhizophagus clarus* extends the possibilities for generating propagules without continuous co-cultivation with a host [5]. Its practical significance depends on the proportion of propagules that remain infective, consistency between batches, storage stability, application dose, and cost. A high spore count does not establish these properties by itself.

A mechanistic extension is provided by myristate-supported cultivation of *R. irregularis*: Sugiura et al. observed hyphal growth and infection-competent secondary spores without a host, with spores smaller than those produced symbiotically [21]. This supports external lipid supply as a route to propagation, but biomass, spore number, and infective units are different manufacturing endpoints. The *R. clarus* production experiment [5] and the *R. irregularis* cultivation experiment [21] should not be ranked solely by raw spore counts because strain, medium, culture duration, and infectivity assays differ.

Host-associated trap or root–organ systems retain a living carbon source but also require management of host biomass, substrate, and contamination. Asymbiotic systems remove continuous host maintenance while adding defined medium, lipid supply, and aseptic-process requirements [3,4,5,21]. The selected sources do not establish a harmonized cost per infective dose or a standardized head-to-head productivity comparison. Consequently, lower production cost and superior industrial scalability are development hypotheses, not demonstrated advantages. Relevant comparisons should report infective units per volume and time, post-storage potency, rejected batches, and cost per usable dose.

Product identity should then be followed beyond manufacturing. Molecular tracking of an introduced *R. irregularis* isolate in *Ziziphus mauritiana* roots illustrates how researchers can investigate whether the applied organism established in the field [6]. Where resident fungi can also colonize the host, an increase in total colonization cannot automatically be attributed to the introduced isolate. A two-year *Medicago sativa* field study examined agronomic responses alongside the persistence of inoculated fungi [7]. Longer follow-up helps determine whether an early response accompanies sustained establishment.

Together, these findings support evaluating identity, viable product, root establishment, persistence, and the final plant outcome as connected but distinct stages. Molecular detection does not prove viability; colonization alone does not establish agronomic value; and a plant response without isolate tracking may be difficult to attribute to the inoculant.

Independent lipid-transfer experiments strengthen the propagation rationale. Jiang et al. demonstrated transfer of host-synthesized fatty acids to *Rhizophagus irregularis* through a RAM2- and lipid-export-dependent pathway [29]. Complementation and lipid-profile experiments further showed how FatM and RAM2 redirect host-lipid metabolism during arbuscular development [30]. Kameoka et al. then identified fatty-acid treatments that stimulated infection-competent secondary spores outside a living host [31]. Together with the myristate experiments [21], these findings connect host metabolic dependence to a cultivation intervention, without implying that one formulation supports all AMF taxa.

Comparative genomes reveal why medium development needs to account for more than carbon supply. The *R. clarus* HR1 genome lacked cytosolic fatty-acid synthase and several other biosynthetic functions [32], and comparisons across *Rhizophagus* and *Gigaspora* identified both shared metabolic losses and divergent gene repertoires [33]. Within *R. irregularis*, substantial isolate-specific gene content [34] and chromosome organization and epigenetic differences [35] reinforce the need to report the strain used in production tests. These genomic findings nominate requirements and sources of variation; they do not establish the optimal composition of a culture medium.

**Figure 2:**
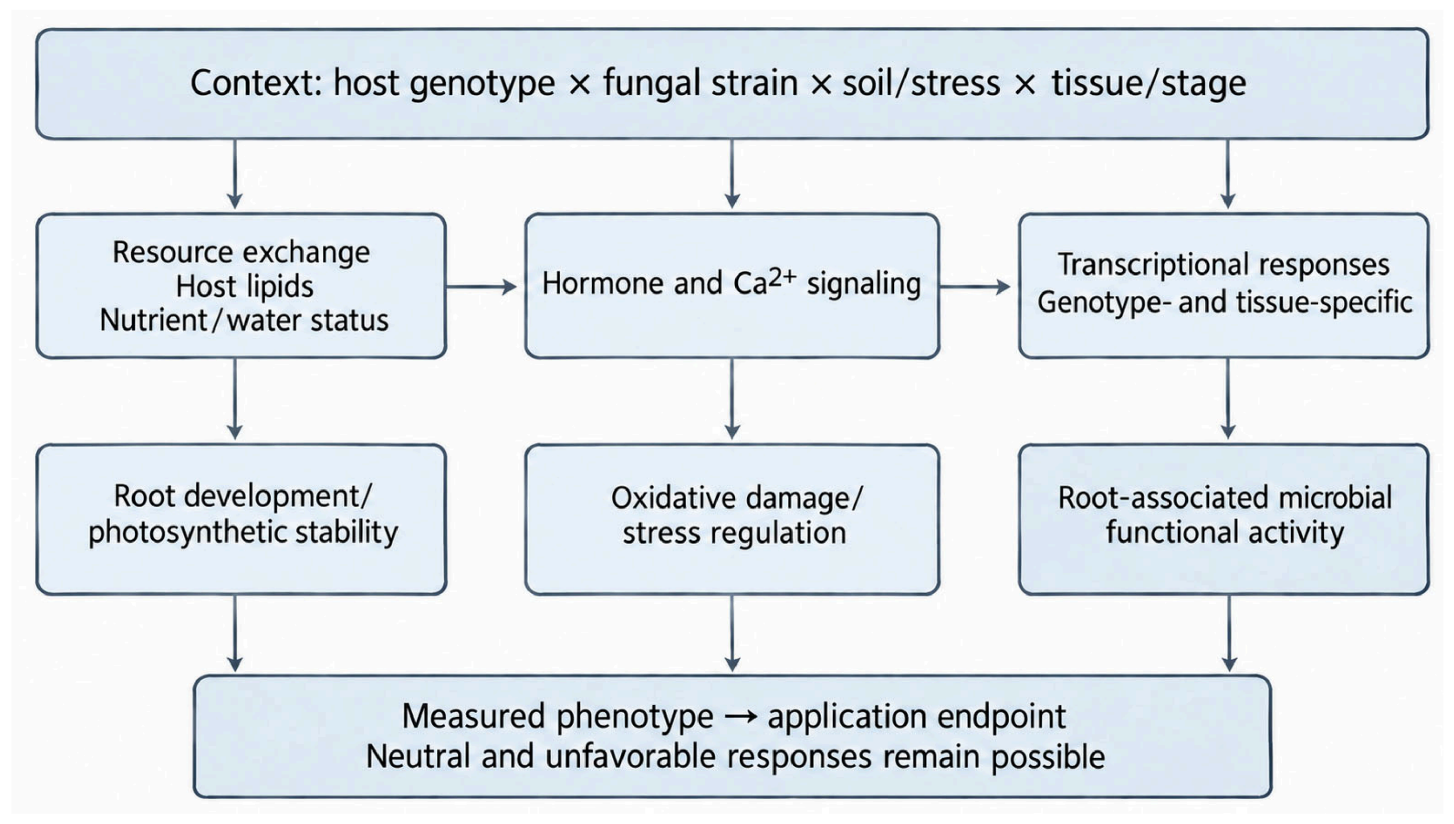
Conceptual integration of molecular and application levels. The diagram links resource exchange [3,4], nutrient [8,11] and hormonal/calcium signaling [23,25,27], transcriptional responses [23,24,25,27], oxidative-stress regulation [25,27], root development [23,24], and root-associated microbial activity [24] to measured phenotypes [2,11,12]. References identify illustrative supporting experiments. Links represent a synthesis across systems, not a complete causal sequence demonstrated in any single study; genotype, fungal strain, tissue, stress, and soil modify the responses.

Identity testing also needs to accommodate marker biology. The discovery of heterogeneous, non-tandem rDNA copies in *R. irregularis* shows that sequence differences within this marker can occur inside one fungal genome [36]. Accordingly, an amplicon variant should not automatically be interpreted as a separate inoculant strain. Root–organ culture optimization has experimentally evaluated medium preparation, gelling agents, inoculum ratio, culture age, harvesting, and drying as determinants of viable propagule output [37]. Separately, excised trap roots supported the formation of *F. mosseae* storage structures [38]. These studies expand the range of production materials, while infectivity assays and storage tests remain necessary before comparing usable doses. Figure 2 summarizes the links between resource exchange, signaling, physiological responses, and measured phenotypes.

### 3.3. Crop Production and Host–Soil Compatibility

Crop inoculation studies need to define what constitutes an effective outcome for the intended production system. Research in cassava linked selection of native arbuscular fungi to growth and yield measurements in greenhouse and field settings [8]. Brazilian maize experiments examined inoculation at different sites and under different phosphorus regimes [9], while wheat studies using inoculants produced on farms reported cultivar-dependent responses [10]. These studies show that the fungus, host, and nutrient-management context cannot be evaluated independently.

The distinction between early growth and harvest performance is important. Increased root colonization or vegetative biomass may support a proposed mechanism, but the final outcome should reflect the harvested crop and its quality. Trials should describe fertilizer management and include a carrier control, together with an appropriate comparison against local phosphorus practice. Otherwise, a response to the formulation or to another input may be attributed incorrectly to the fungus.

Variation among fields is a major obstacle to generalization. In 54 Swiss maize fields, plant growth responses to arbuscular mycorrhizal inoculation ranged from −12% to +40%; soil microbiome indicators explained part of this heterogeneity [2]. Positive responses in some fields are therefore compatible with weak or unfavorable responses elsewhere. A useful next step is to identify measurable site characteristics that predict where inoculation is likely to help.

Compatibility also varies within a host–fungus system. In rice, combinations of cultivar and fungal genotype differed in growth and mycorrhiza-induced resistance [11]. In sorghum, contrasting arbuscular fungal associations were accompanied by different root metabolic profiles and beneficial or unfavorable plant responses [12]. These results do not support a fixed beneficial effect based solely on a fungal species name. Selection should focus on defined strain–host–soil combinations, followed by validation under the range of conditions in which use is proposed.

The crop evidence supports a conditional application model. Field trials should record host genotype, soil properties, resident microbiota, and nutrient management; prespecify a harvest-relevant primary outcome; and report absent or negative responses as well as gains. This reporting is necessary to identify circumstances in which inoculation offers a repeatable advantage.

Wheat experiments bridge mechanistic measurements and harvest-related outcomes. An integrated transcriptomic, proteomic, and metabolomic study reported greater grain yield and reduced lesions caused by *Xanthomonas translucens* in mycorrhizal plants [39]. A separate assessment of 94 wheat genotypes identified variation and quantitative trait loci associated with root colonization [40]. The latter measures genetic control of establishment, whereas the former measures particular performance outcomes; colonization loci should therefore not be labeled yield-benefit loci without a direct test. In rice, variation at OsCERK1 linked symbiotic competence to phosphorus acquisition and agronomic traits in the tested genetic materials [41], illustrating a route for host selection alongside fungal selection.

Within field-grown maize, root type and soil phosphate altered fungal assemblages and host transcription [42]. Across farms, greater crop diversity enriched AMF communities, although root colonization did not differ consistently between management systems [43]. These observations distinguish management of indigenous symbioses from addition of a commercial strain: a technology can support existing communities through crop and nutrient management, and increased community richness cannot be substituted for a measured inoculation benefit.

Phosphorus mobilization can involve a fungal–bacterial partnership rather than an enzyme supplied directly by AMF. Field hyphal-ingrowth cores linked enhanced organic-phosphate mineralization to bacteria associated with fungal hyphae [44]. Microcosm and in vitro experiments demonstrated transport of phosphate-solubilizing bacteria along hyphae [45], while a defined *R. irregularis*–*Rahnella aquatilis* system showed that fungal fructose exudation promoted bacterial phosphatase-related responses and phytate mineralization [46]. These studies retain the arbuscular component within scope because they investigate its identifiable contribution. Their results support testing functional consortia, with controls able to separate fungal, bacterial, and combined effects.

### 3.4. Molecular Mechanisms Underlying AMF-Mediated Plant Performance

The molecular evidence links successful establishment to resource allocation and host regulation rather than to a universal beneficial program. Host-lipid biosynthesis and transfer sustain fungal development [3,4], while rice cultivar–fungal genotype and sorghum-metabolome experiments show that establishment can accompany contrasting host outcomes [11,12]. A larger absorptive interface or altered nutrient status can influence the phenotype, but improved performance cannot be assigned to nutrient uptake without measuring the relevant resource flux or plant nutrient status. Figure 2 therefore connects mechanisms and phenotypes while retaining the possibility of neutral or unfavorable outcomes.

Virág et al. compared two maize inbred lines differing in drought tolerance and their hybrid after inoculation with *F. mosseae* BEG12 under controlled water regimes [23]. The tolerant line largely maintained photosynthetic stability, whereas the sensitive line displayed greater root plasticity and hormone-associated transcriptional changes; the hybrid followed a distinct response. These findings provide direct evidence that genotype changes which processes respond to an inoculant. They support targeted compatibility testing rather than a fixed claim of drought protection for all maize plants.

A complementary root-focused study by Virág et al. linked contrasting drought responses to genotype-dependent transcriptional reprogramming and functional activity in non-host root-associated transcripts [24]. Stress regulation, metabolic adjustment, and root remodeling differed among inbred lines and the hybrid. Microbial transcript activity provides information beyond community membership, but it does not prove that a specific microbial change caused improved plant performance. The two reports share authors and closely related maize systems, so they offer complementary resolution rather than broad independent replication across crops or environments.

Tissue is another source of heterogeneity. Szentpéteri et al. used root and leaf RNA-seq in heat-stressed tomato associated with *Septoglomus constrictum* [25]. Root-hair development and stress-related responses differed from leaf auxin transport and ethylene-related pathways. Lower hydrogen peroxide and malondialdehyde accompanied altered stress-gene expression, linking transcriptional observations to oxidative-damage measurements. Roots and leaves therefore cannot be treated as interchangeable sources of a stress-response signature. Nevertheless, pathway enrichment and concurrent physiological change do not by themselves establish the causal contribution of each gene.

A maize experiment with *F. mosseae* also combined root and leaf RNA-seq with calcium measurements and identified tissue-preferential expression within calcium-signaling networks, including CBL/CIPK, MAPK, and CDPK-related genes [27]. This extends the mechanistic bridge from nutrient and hormonal context to transcriptional regulation and physiology. Such expression and co-expression associations nominate pathways for perturbation studies; they should not be interpreted as proof that each identified regulator is necessary or sufficient for drought tolerance.

Across these studies, host genotype, fungal strain, stress intensity, soil context, developmental stage, and sampled tissue define the response being evaluated. A transcriptomic signature becomes a candidate predictive marker only after independent validation links it to an application endpoint across those conditions. Useful next steps combine gene expression with root architecture, nutrient uptake, water status, oxidative-damage measurements, and harvested output, while explicitly testing genotype × inoculation × environment interactions.

Comparative plant genomic and transcriptomic analyses place AMF signaling within an ancestral intracellular-symbiosis pathway [47]. At the experimental level, combined recognition of chitooligosaccharides and lipochitooligosaccharides promoted symbiotic signaling and balanced immunity in *Medicago truncatula* [48]. In rice, OsMYR1/OsLYK2 and OsCERK1 formed a receptor complex involved in signal perception [49], and OsCERK1 knockout plants lost the nuclear calcium response to short-chain fungal chitin signals [50]. This receptor and calcium evidence is more directly mechanistic than differential gene expression alone, but the conserved signaling capacity does not imply equal crop responses to inoculation.

Colonization is also actively regulated by host resource status. CLE peptides acting through SUNN modulated strigolactone content and fungal colonization [51], whereas CBX1 coordinated expression of host phosphate-uptake and lipid-biosynthesis genes in *Lotus japonicus* [52]. Nutrient starvation regulated lipochitooligosaccharide recognition through NSP1 and NSP2 [53]. A Medicago SWEET transporter contributed to arbuscule maintenance, but the absence of a clear defect in single loss-of-function mutants indicated functional redundancy [54]. These results caution against assigning symbiotic control to a single universally predictive gene or assuming that more colonization is always desirable.

Transport studies provide a concrete bridge between an association and resource acquisition. Functional characterization of OsNPF4.5 identified a mycorrhizal nitrate-uptake route in rice [55]. In maize, ZmAMT3;1 localization, heterologous transport assays, knockdown lines and compartmented experiments supported a mycorrhiza-dependent ammonium pathway, with a contribution to postsilking nitrogen acquisition also measured under field conditions [56]. Such designs establish transport function more strongly than total shoot nitrogen alone. Nevertheless, pathway contributions remain conditional on nitrogen form, supply, and developmental stage and should not be treated as a general fertilizer-replacement percentage.

Phosphorus and potassium acquisition are likewise host regulated. Radiotracer experiments across maize lines linked mycorrhizal phosphorus uptake with extraradical hyphal development and PHT1 transcript accumulation [57]. In tomato, loss- and gain-of-function tests implicated SlHAK10 in the mycorrhizal potassium pathway under low-potassium conditions [58]. The practical implication is to measure the limiting nutrient and the relevant uptake route, rather than infer a common nutrient mechanism from biomass responses across different crops.

Fungal resource delivery is spatially and temporally dynamic. Quantum-dot tracking showed phosphorus redistribution across networks exposed to unequal resource patches [59] and changes in allocation associated with the demand of different root hosts [60]. These experiments resolve network behavior in controlled systems; they do not quantify a field-wide yield gain. Hyphosphere metagenomics and compartmented Medicago experiments linked fungal recruitment of bacteria to organic-phosphorus turnover [61], while field and glasshouse comparisons identified recurring hyphosphere bacterial groups contributing to phosphatase activity [62]. This evidence complements the transport studies by locating part of nutrient mobilization in the associated microbial community.

#### Drought Salinity and Temperature Responses

Water-transport measurements clarify a mechanism often inferred from aquaporin expression. Maize root-cell assays showed maintenance of water permeability and hydraulic conductivity in droughted mycorrhizal roots, together with altered aquaporin phosphorylation [63]. Tomato pot experiments comparing *F. mosseae* and *R. intraradices* demonstrated fungal differences in water-stress responses [64]. In common bean, laser microdissection and transcript profiling resolved drought-related changes within arbuscule-containing cells [65]. In contrasting maize cultivars, improved drought performance accompanied different patterns of aquaporin regulation, including downregulation of several genes in the sensitive cultivar [66]. A beneficial response therefore need not involve uniform aquaporin upregulation.

Metabolic plasticity adds another level of strain dependence. Tomato comparisons across three fungal partners revealed both shared and isolate-specific metabolic changes under drought or salinity, with stress severity modifying the response [67]. Apple experiments associated improved drought physiology with antioxidant activity, osmotic adjustment, and MAPK-related transcription [68]. These observations support integrated physiological and molecular assessment, but do not establish that pathway activation alone causes tolerance or predicts a repeatable harvested output.

Salinity studies separate oxidative protection from ion homeostasis. Cucumber inoculation altered antioxidant systems, hormones, and mineral profiles while reducing sodium accumulation [69]. In lettuce, two inocula and two cultivars differed in colonization and antioxidant responses; improved growth could accompany lower phenolic accumulation and less oxidative damage [70]. Black-locust experiments linked photosynthetic and water-status responses to K+/Na+ balance and expression of ion-transport genes [71]. Thus, higher phenolic content is not a universal signature of protection, and sodium exclusion, nutrient balance, and tissue damage should be measured alongside growth.

Responses under actual water limitation can weaken as stress intensifies. An in situ rainfall-exclusion study found different AMF-associated responses in C3 and C4 grasses, with fewer physiological benefits maintained under the most severe drought [72]. Combined drought and heat experiments in tomato showed stronger protection with *S. constrictum* than with *S. deserticola* and different responses across stress combinations [73]. Evidence from separate single-stress assays should consequently not be added together to predict performance under multiple stresses.

Temperature experiments further connect photosynthesis to conditional protection. Maize pot studies measured improved photosystem performance under high temperature [74], whereas low-temperature transcriptomics identified changes in photosynthetic electron flow and respiratory metabolism [75]. A multispecies AMF inoculum altered physiological and metabolomic responses of peanut in separate salt, drought, and cold treatments [76]. Because the peanut treatment was a fungal mixture, its outcome cannot identify the most effective constituent strain. None of these experiments establishes a universal temperature range for commercial efficacy.

### 3.5. Phytomanagement of Contaminated Soil

Arbuscular mycorrhizal fungi have been investigated where contaminant exposure constrains plant establishment. Experiments with *Diversispora eburnea* in cadmium-contaminated soil measured growth, cadmium uptake, and changes in soil metal speciation [13]. Studies of *Rhizophagus irregularis* in alfalfa examined metal accumulation together with responses involving transporter and chelator-related genes [14,15]. These measurements inform possible biological processes, but they address different questions and should not be treated as interchangeable indicators of remediation.

The cadmium evidence illustrates why mechanism and management target must be interpreted together. In alfalfa, *R. irregularis* reduced shoot cadmium concentration and was associated with lower expression of MsPCS1, MsMT2, MsIRT1, and MsNramp1; fungal sequestration was proposed as an explanation rather than directly established as the sole cause [14]. Reduced plant exposure can therefore coincide with lower induction of transporter and chelator genes, rather than their universal activation. In a different plant–soil system, *D. eburnea* increased exchangeable cadmium while reducing the residual fraction, accompanying greater root accumulation [13]. These contrasting responses support context-specific retention or mobilization pathways, not a general claim that AMF always immobilizes metals.

Fungal binding or sequestration, altered plant transport, chelation, and changes in soil-chemical availability are distinct candidate processes. To assign their relative contributions, experiments would need compartment-resolved metal measurements or tracer/perturbation designs. Expression of a plant transporter is an intermediate response; it does not locate the metal inside fungal structures or establish contaminant removal. Likewise, root accumulation is not phytoextraction unless that contaminated biomass is recovered and managed. These distinctions constrain interpretation of the transporter and tolerance responses reported in alfalfa [14,15].

The first decision in evaluating an intervention is the site-level aim. A treatment that supports plant cover may be useful for stabilization without increasing extraction. Conversely, greater cadmium uptake may be relevant to removal when the contaminated biomass is harvested and safely managed. The desired direction of a change depends on the intended strategy.

For phytostabilization, the endpoint concerns contaminant mobility or exposure. For phytoextraction, it includes the total contaminant mass removed in harvested biomass. Where food crops are involved, the edible tissue must be assessed separately. A larger plant can have a lower tissue concentration merely because an absorbed metal is distributed across more biomass; this does not prove that the soil stock decreased. Studies should therefore report both tissue concentration and biomass, calculate total content when appropriate, and examine relevant soil chemistry.

Differences in host species, fungal isolate, soil composition, and exposure level limit direct comparisons between experiments. Evidence that inoculation modifies gene expression in one system cannot establish the scale of contaminant removal at another site. Monitoring over a period suited to the remediation goal is needed before recommending a *Glomeromycota*-based intervention.

Direct localization provides stronger support for fungal metal retention than transporter expression alone. In *Lotus japonicus*, stable-isotope tracing and synchrotron X-ray fluorescence localized hyphally acquired cadmium within intraradical fungal structures [77]. This demonstrates a retention mechanism in that system. In contrast, ryegrass experiments showed increased cadmium uptake and accumulation alongside improved tolerance, altered ion influx, and subcellular sequestration [78]. These contrasting outcomes explain why phytostabilization and phytoextraction require different endpoints even when both treatments increase plant growth.

Metal identity and crop stage also change interpretation. Arsenic experiments in alfalfa measured altered speciation, partitioning, and phosphorus-related gene expression [79]; they should not be reduced to the cadmium model. In rice, cadmium-chemical forms and grain exposure differed across growth stages and soil concentrations [80]. Soybean responses further varied with genotype and included changes in nutrient partitioning and transporter expression [81]. A mitigation claim for an edible crop needs measurements in the harvested tissue at maturity, rather than an assumption based on early root sequestration.

Subcellular distribution in *Phragmites australis* [82] and photosynthetic responses in hemp [83] supply complementary tolerance endpoints, but neither endpoint alone establishes net soil decontamination. In *Broussonetia papyrifera*, cadmium influx, redox regulation, and metal-transport-related transcription changed differently across contamination levels [84]. The same fungus can therefore engage different retention or uptake responses as exposure increases. Application assessment should specify contaminant loading, plant use, biomass disposal and the fate of the residual soil pool.

#### Organic Pollutants and Compatibility with Chemical Management

Pollutants may impair the proposed biological technology as well as provide its remediation target. A trans-European soil survey coupled to a greenhouse radiotracer assay associated intensive agricultural management and fungicide use with reduced AMF-mediated phosphorus transfer [85]. A pesticide-cocktail experiment detected soil- and taxon-dependent community responses [86], while glyphosate treatment reduced mycorrhizal colonization and grass growth in a field-pot design, with effects conditioned by tillage and cultivation history [87]. Compatibility with a local pesticide program should therefore be tested rather than assumed from a successful pesticide-free inoculation assay.

AMF-associated degradation of organic contaminants can be indirect. In a factorial hyphal-compartment experiment, fungal hyphae and earthworms accelerated oxytetracycline degradation and altered bacterial assemblages; degradation intermediates were also identified [88]. This supports a hyphosphere-mediated intervention, rather than proof of a purified fungal degradative enzyme. A remediation evaluation still needs residual parent compound, metabolites, toxicity, and persistence measurements to determine whether disappearance of the parent pollutant yields a safer site.

### 3.6. Plant Metabolites and Their Technological Interpretation

Mycorrhizal associations may influence plant-produced compounds, but the response can depend on tissue and sampling time. In *Anchusa officinalis*, experiments with *Rhizophagus irregularis* examined shoots, roots, and exudates in a semi-hydroponic system; the observed metabolic differences varied between experimental periods and sample types [16]. A change detected in an exudate at one time should not be assumed to characterize a harvested shoot or root at another.

Work with *Glycyrrhiza uralensis* under drought examined accumulation of glycyrrhizin and liquiritin [17]. Experiments in *Alkanna tinctoria* investigated alkannin/shikonin-related compounds across cultivation systems [18]. Brazilian studies evaluated leaf biomolecules in *Passiflora edulis* and secondary metabolites in *P. setacea* following inoculation with arbuscular fungi [19,20]. Together, these experiments identify promising plant–fungus combinations but do not demonstrate that the same response will occur with other cultivars, isolates, or production practices.

The origin of the measured substance must be stated accurately. In these examples, the compounds are produced or accumulated by the plant under the influence of the fungal association. A change after inoculation does not make them direct fungal products; demonstrating fungal production would require a design able to separate the plant and fungal sources.

For an industrial application, concentration is only an initial result. The amount recovered per plant, harvest, or production area also depends on biomass, sampled tissue, and extraction yield. Development would require confirmation of chemical identity, quantified total output, consistency between batches, and feasibility at the intended cultivation scale. Altered accumulation of a compound associated with medicinal use does not establish clinical efficacy or safety.

Plant metabolic regulation is not always explained by improved phosphorus nutrition. In *Medicago truncatula*, comparison with high-phosphate fertilization distinguished mycorrhiza-associated hormonal and flavonoid/terpenoid responses from changes shared with nutrient supplementation [89]. A two-year thyme–soybean intercropping experiment measured essential-oil quantity and composition across water regimes and fungal treatments [90]. These designs strengthen application-specific attribution, while also showing that cropping arrangement and water supply belong in the production specification.

Chemical responses can include decreases as well as increases. Trifoliate-orange metabolomics found declines in most differential terpenoids, with a contrasting drought-associated increase in beta-caryophyllene [91]. In fruit-bearing grapevine cuttings, anthocyanin responses accompanied changes in abscisic-acid metabolism under temperature and irrigation treatments [92]. These observations argue for a target compound and tissue defined before analysis, with chemical identity and quantity verified independently of an untargeted pathway annotation.

An open-field comparison of tarragon, lavender, and hyssop reported increased essential-oil content but species-dependent growth responses and no difference in antioxidant activity or phenolics at harvest; storage conditions changed the interpretation [93]. In olive roots, drought and *R. irregularis* altered the balance of oleuropein, verbascoside, and mannitol [94]. Useful chemical output is therefore not synonymous with stronger antioxidant activity, and a root metabolite shift does not establish a higher yield in the harvested organ used by an industrial process.

#### Defense Responses and Attribution in Microbial Consortia

Biocontrol-related studies extend the chemical discussion toward a functional plant endpoint. Tomato mosaic virus experiments measured disease and viral accumulation alongside changes in phenylpropanoid-related transcription and flavonoids [95]. Root transcriptomics under water deficit and nematode infection identified responses to simultaneous biotic and abiotic challenges [96], while Medicago receptor mutants demonstrated a dual role for MtLYK9 in AMF colonization and immunity against an oomycete [97]. These results connect perception and defense to particular pathosystems; they do not demonstrate a broad-spectrum fungal antimicrobial preparation.

A strawberry factorial study of three AMF and three *Pseudomonas* strains measured plant development, fruit composition, and volatiles and attributed several fruit-production responses primarily to the bacterial partner [98]. It is therefore inappropriate to label every benefit of a combined inoculum an AMF effect. Consortia may be technologically useful, but the final product claim should specify the tested combination and preserve separate fungal, bacterial, and combined controls.

### 3.7. Comparative Evidence and Priorities

The selected studies occupy different points between biological discovery and routine use. Lipid-transfer experiments explain constraints on propagation [3,4]; asymbiotic production addresses manufacturing [5]; fungal tracking investigates establishment [6]; and crop trials test outcomes under variable conditions [2,7,8,9,10,11,12]. Studies of contaminated soil and plant metabolites require endpoints specific to their intended applications [13,14,15,16,17,18,19,20]. A positive result at one stage does not complete the evidence needed at the next.

Across applications, a product should be described by isolate identity, viable dose, formulation, and storage conditions. Controls should distinguish fungal effects from carrier, fertilizer, or other treatment components. Tracking the introduced fungus can strengthen attribution where resident fungi occur. The primary outcome should match the proposed use: harvested crop output, contaminant fate, or chemically verified compound yield.

Table 2 distills the translational questions raised by Table 1. An application reaches a stronger level of evidence when the fungal identity is clear, the controls isolate the fungal contribution, and the endpoint matches the proposed use. Multiple sites are particularly informative for agricultural deployment; contaminant studies need a defined exposure or mass-balance endpoint; and metabolite studies require total chemical yield and repeatability. These criteria distinguish a plausible mechanism or proof of concept from a candidate practice ready for external validation.

#### Product Quality Industrialization and Ecological Monitoring

Commercial quality cannot be inferred from propagation feasibility. Salomon et al. evaluated commercial inoculants across greenhouse and field conditions and found inconsistent viable-propagule performance; in non-sterile greenhouse soil, biomass increased in only one of 25 treatments [22]. Boussageon et al. separately compared 16 agricultural/home-gardening products and seven research products; only three of the 16 agricultural/home-gardening products induced both colonization and a positive growth response [28]. These product-specific evaluations support independent potency testing, but their denominators should not be pooled or generalized to every manufacturer.

Industrial evaluation should distinguish identity, infective dose, shelf-life potency, carrier composition, contamination control, and final efficacy. The selected research supports the need for verification, but does not establish universally accepted acceptance thresholds or a harmonized international regulatory standard [5,6,22,28]. A product dossier should state its jurisdiction and intended claims and determine applicable requirements rather than assume that all bioinoculants are regulated alike. Comparative manufacturing economics remain a gap: laboratory propagation success is insufficient to demonstrate affordability after formulation, storage, transport, and field application.

Resident microbiota can supply similar functions, inhibit establishment, or alter the host response, making inoculation in biologically active soil different from a sterilized-substrate assay [2,22]. Islam et al. followed a commercial *R. irregularis* inoculant across four Canadian sites for three cropping seasons; detectability persisted for 27 months at two sites but ended earlier at the others [26]. Site-dependent persistence makes ecological monitoring relevant, yet persistence alone is not proof of invasion or biodiversity loss. Before deployment outside a strain’s source setting, monitoring should examine off-target spread, changes in indigenous AMF composition and function, and the persistence of those changes. Locally sourced candidates can be compared with commercial strains without presuming that local origin guarantees safety or superior efficacy.

Revegetation provides an additional setting for evaluating ecological compatibility. Inoculation of native shrub species altered rhizosphere bacterial assemblages alongside host growth, with host identity conditioning the response [99]. Such community shifts establish an ecological effect, not necessarily harm or improved long-term ecosystem function. Restoration trials should measure plant establishment and persistence together with resident-community trajectories and soil outcomes.

Carbon and water outcomes can diverge from a simple beneficial narrative. A carbon-labeling experiment found contributions of AMF communities to mineral-associated organic carbon while some community treatments also decreased total soil organic carbon [100]. In root-excluding soil compartments, *R. irregularis* altered water retention and hydraulic conductivity in opposite directions in sand and loam [101]. These experiments show why total carbon balance and soil texture must be considered before claiming carbon sequestration or improved water storage.

A global survey and niche-modeling study identified temperature and pH as major predictors of AMF distributions [102]. This is observational evidence for environmental filtering, not a field test of inoculant performance. Together with strain-specific genomes, host signaling and field heterogeneity, it supports testing environmental compatibility at the intended deployment site rather than assuming that widespread occurrence guarantees local functionality.

Table 2 distinguishes experimentally supported assessment needs from author-proposed development criteria. Its evidence descriptors indicate where support originates, not a validated checklist or formal confidence grade.

**Figure 3:**
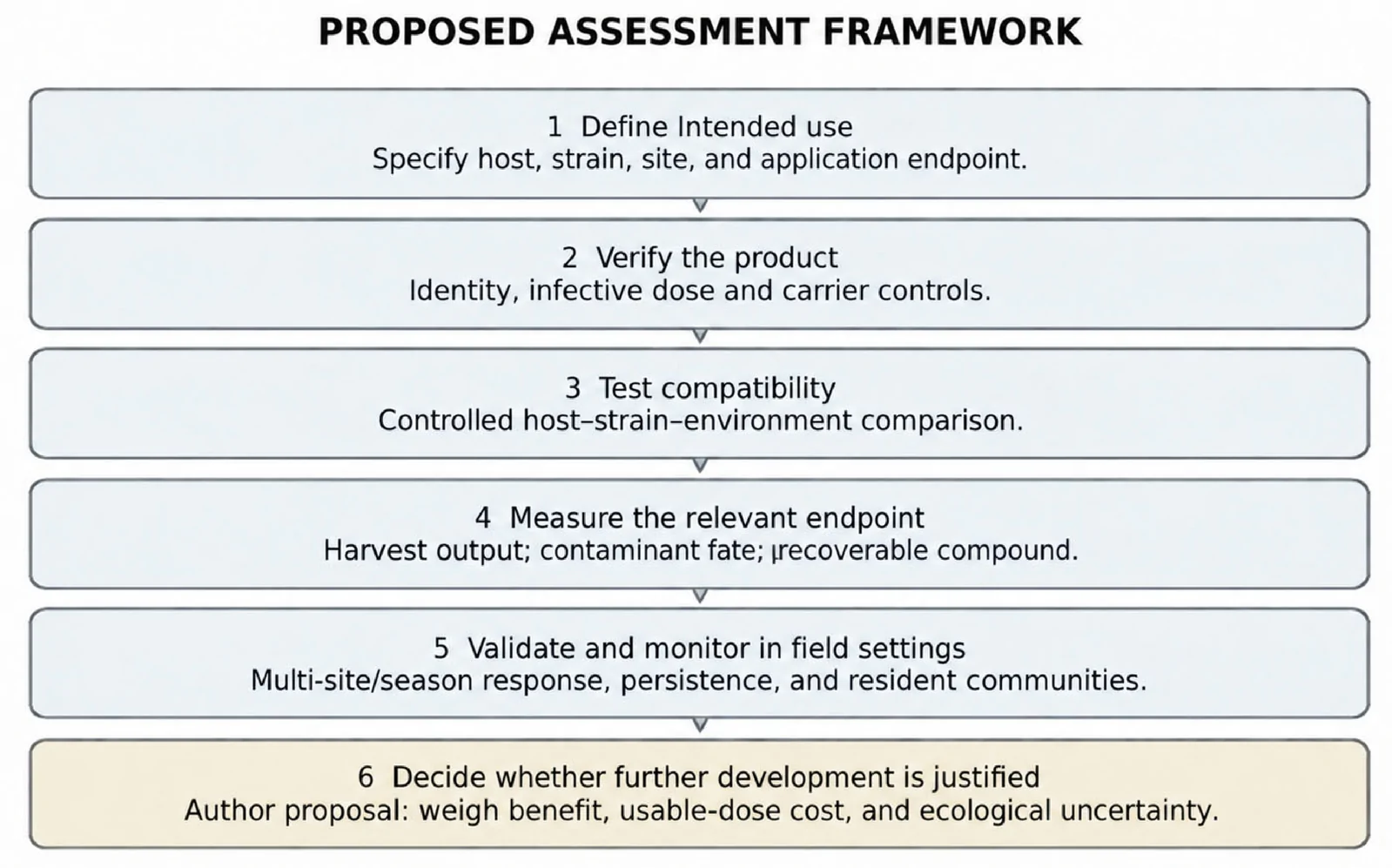
Author-proposed framework for evaluating *Glomeromycota* inoculants. References support individual assessment questions: intended use [2,8,9,10,11,12], product verification [5,6,21,22,28], compatibility [11,12,23,24,25,27], relevant endpoints [7,8,9,10,11,12,13,14,15,16,17,18,19,20], and field validation and monitoring [2,6,7,26]; the ordered sequence and advancement decisions are methodological recommendations. The framework has not been prospectively validated, is not a regulatory standard, and does not define universal numerical acceptance thresholds.

Figure 3 likewise organizes questions for future testing; its ordering and advancement decisions have not been prospectively validated, but provide a framework for evaluating *Glomeromycota* inoculants.

### 3.8. Bioactive Compounds and Limits of Attribution

The categories used to describe bioactive compounds from culturable fungi cannot be transferred unchanged to an arbuscular mycorrhizal review. The studies analyzed here primarily test a fungal association and a plant response. When an inoculated plant accumulates a phenolic compound, an organic acid, or a colored specialized metabolite, the measured substance remains plant-derived unless its fungal origin is established separately. Table 3 distinguishes direct evidence in the selected studies from related findings that address another biological question.

The chemical evidence concerns plant compounds and their regulation across tissues, stresses, cultivation systems, and harvest conditions [16,17,18,19,20,89,90,91,92,93,94]. Antioxidant responses and plant disease suppression [20,95,96,97] are distinct from isolating an antioxidant or antimicrobial produced by the fungus. Claims about fungal natural products require fungal origin, chemical identity, recoverable yield, and an appropriate functional assay to be established separately.

Table 4 compares culture approaches and makes the absence of standardized productivity and economic comparisons explicit.

## 4. Conclusions and Future Directions

*Glomeromycota* biotechnology should be developed around defined host × fungal strain × environment combinations, with tissue and developmental stage specified when interpreting molecular or chemical responses. Lipid-transfer and cultivation experiments support propagation research; transcriptomic studies explain why the same inoculation can engage different photosynthetic, root-development, hormonal, calcium-signaling, and stress-response processes. Those mechanisms help generate testable hypotheses but do not establish repeatable harvest benefits. Field heterogeneity, unreliable commercial propagule quality, and variable persistence require attention alongside controlled efficacy. Colonization is an establishment endpoint, not proof of agronomic value; biomass gain is not proof of contaminant removal; and plant chemical modulation is not evidence of fungal biosynthesis or therapeutic efficacy. Priorities are independently verified for product identity and infectivity, comparable cost-per-infective-dose measurements, multi-site and multi-season validation, and monitoring of introduced strains and resident communities. The proposed framework makes these questions explicit but remains unvalidated. Because this narrative set is selective, a broader independently screened literature assessment is still needed before making global coverage claims or quantitative recommendations.

## Figures and Tables

**Table 1:** Detailed evidence map for 28 illustrative original studies within the 102-article narrative synthesis. Each row links one article to its fungus or inoculum, verification information, host and setting, and measured endpoint. NR indicates information not established from the source material checked.

Study	Fungus or Inoculum	Identification/Verification Reported	Host and Setting	Application or Measured Outcome
Rosling et al. [1]	Multiple *Glomeromycota* lineages	Phylogenomic comparison; not an inoculant identity assay	Comparative genomes	Genomic signatures of obligate arbuscular symbiosis.
Lutz et al. [2]	Arbuscular mycorrhizal inoculum; species NR in abstract	Taxonomic-identification method NR in abstract	Maize; 54 fields	Variable growth response; soil-microbiome indicators.
Luginbuehl et al. [3]	Arbuscular mycorrhizal fungi; species NR in abstract	Taxonomic-identification method NR in abstract	Plant–fungus experimental system	Host-derived fatty acids inform propagation.
Liu et al. [4]	*R. irregularis*; *F. mosseae*	Named experimental fungi; taxonomic method NR in abstract	Rice; molecular experiment	Host OsRAM2 and symbiotic lipid exchange.
Tanaka et al. [5]	*R. clarus* HR1	Named isolate; spore infectivity assessed; taxonomic method NR	Asymbiotic culture; host assay	Mass production of infection-competent spores.
Thioye et al. [6]	*R. irregularis* IR27	Isolate-specific RPB1 qPCR for root tracking	Ziziphus; field	Tracks the applied isolate after inoculation.
Pellegrino et al. [7]	*F. mosseae* BEG12/AZ225C; *R. irregularis* BEG141; mixtures	Named isolates; root persistence assessed; taxonomic method NR	Alfalfa; two-year field trial	Yield, forage nutrients/fatty acids, persistence.
Séry et al. [8]	*Acaulospora colombiana* LBVM01; *Ambispora appendicula* LBVM02	LSU–ITS–SSU PCR products analyzed phylogenetically	Cassava; greenhouse and field	Selection of native fungi for growth and yield.
Stoffel et al. [9]	*R. intraradices* as described in the original article	Commercial inoculant; taxonomic method NR in available text	Maize; multiple Brazilian sites	Yield/biomass under different phosphorus regimes.
Fornari et al. [10]	On-farm arbuscular inoculants; species NR in available text	Taxonomic identification method NR in available text	Wheat; cultivars	Cultivar-dependent responses to farm-produced inocula.
Guigard et al. [11]	*F. mosseae*; *R. irregularis*	Named fungal genotypes; taxonomic method NR in abstract	Rice; cultivar comparisons	Growth and induced-resistance compatibility.
Kaur et al. [12]	*R. irregularis*; *Gigaspora gigantea*	Named inocula; taxonomic method NR in abstract	Sorghum; controlled experiments	Mutualistic or unfavorable growth/metabolome.
Sun et al. [13]	*Diversispora eburnea*	Named inoculum; taxonomic method NR in abstract	Lolium; Amorpha; Cd soil	Growth, Cd uptake, and soil Cd speciation.
Motaharpoor et al. [14]	*R. irregularis*	Named inoculum; fungal ID method NR in abstract	Alfalfa; Cd exposure	Cd uptake and plant transporter/chelator genes.
Wang et al. [15]	Arbuscular fungus; species NR in abstract	Taxonomic-identification method NR in abstract	Alfalfa; Cd exposure	Cd transport and stress tolerance.
Cartabia et al. [16]	*R. irregularis* MUCL 41833	Named collection strain; taxonomic method NR in abstract	Anchusa; semi-hydroponics	Plant tissue and exudate metabolite profiles.
Xie et al. [17]	Arbuscular fungus; species NR in abstract	Taxonomic-identification method NR in abstract	Glycyrrhiza; drought	Plant glycyrrhizin and liquiritin accumulation.
Zhao et al. [18]	*R. irregularis* MUCL 41833; *R. aggregatus* MUCL 49408; native *R. irregularis* and *Septoglomus viscosum*	Named collection/native strains; fungal ID method NR in abstract	Alkanna; hydroponics and pots	Plant alkannin/shikonin-related compounds.
de Oliveira et al. [19]	*Acaulospora longula*; *Claroideoglomus etunicatum*; *Gigaspora albida*	Named inocula; taxonomic method NR in abstract	*Passiflora edulis*; seedlings	Leaf vitexin, flavonoids, and saponins.
Muniz et al. [20]	*Gigaspora albida*	Named inoculum; taxonomic method NR in abstract	*Passiflora setacea*; seedlings	Leaf phenols, tannins, saponins, and antioxidant activity.
Sugiura et al. [21]	*R. irregularis*; *R. clarus*; *G. margarita*	Growth and secondary-spore infectivity measured	Defined-lipid asymbiotic culture	Myristate supports growth; infective secondary spores.
Salomon et al. [22]	Commercial AMF formulations	Product potency/colonization assessed; composition varies	Greenhouse and field evaluations	Quality and efficacy vary; no biomass benefit in most tested treatments.
Virág et al. [23]	*F. mosseae* BEG12	Named accession; colonization and RNA-seq	Two maize inbred lines and hybrid; drought pots	Genotype-specific photosynthetic/root/hormonal responses.
Virág et al. [24]	*F. mosseae*	Colonization; root RNA-seq and non-host transcripts	Maize lines and hybrid; greenhouse drought	Root reprogramming and microbial activity depend on genotype.
Szentpéteri et al. [25]	*S. constrictum*	Named inoculum; root/leaf RNA-seq	Tomato; controlled heat stress	Tissue-specific pathways and oxidative-damage responses.
Islam et al. [26]	Commercial *R. irregularis*	Molecular detection and community phylogeny	Four Canadian sites; three crop seasons	Persistence differs across sites; resident-community assessment.
Zhang et al. [27]	*F. mosseae*	RNA-seq and Ca2+ content/flux measurements	Maize roots and leaves; drought pots	Tissue-preferential calcium-signaling gene expression.
Boussageon et al. [28]	Sixteen commercial and seven research AMF products	Propagules, colonization and growth tested	Controlled product comparison	Only 3/16 commercial products colonized roots and improved growth.

**Table 2:** Assessment needs linked to selected evidence. M, C, and F describe mechanistic, controlled, and field settings. Unestablished requirements are identified explicitly; no formal GRADE score or validated checklist is claimed.

Assessment Question	Supporting Selected Evidence	Evidence Scope and Remaining Requirement
Identity and attribution	Named inocula, tracking, and genomic marker variation [5,6,7,8,26,32,33,34,35,36]	M/F: tracking supports attribution; identity and viable dose remain separate.
Infective potency and product quality	Infective spores, culture optimization, and product tests [5,21,22,28,31,37,38]	C/F: potency testing supported; universal dose/shelf-life thresholds not established.
Production efficiency and cost	Defined-lipid propagation and root–organ optimization [5,21,31,37]	C: feasibility demonstrated; normalized cost per usable dose and head-to-head productivity not established.
Host and environmental compatibility	Multi-field/cultivar studies, and root/genotype effects [2,7,8,9,10,11,12,39,40,41,42,43]	C/F: context dependence supported; reproducibility needs multi-site/season testing.
Molecular stress responses	Genotype/tissue studies, transport, and stress physiology [23,24,25,27,47,48,49,50,51,52,53,54,55,56,57,58,59,60,61,62,63,64,65,66,67,68,69,70,71,72,73,74,75,76]	M/C: pathways associated with response; causal regulators and predictive biomarkers need independent tests.
Contaminant management	Metal localization, uptake, and pollutant experiments [13,14,15,77,78,79,80,81,82,83,84,85,86,87,88]	M/C: localization strengthens some mechanisms; mass removal and stable exposure reduction still need direct measurement.
Chemical production	Plant chemistry and defense experiments [16,17,18,19,20,89,90,91,92,93,94,95,96,97,98]	C: chemical modulation; verified total recoverable yield and repeatability still needed.
Ecological compatibility	Persistence, field heterogeneity, and soil/community outcomes [2,6,26,99,100,101,102]	F: site-dependent establishment; off-target spread and long-term effects are proposed monitoring needs.

**Table 3:** Evidence map for chemical and other biotechnological claims in the 102 selected studies. Absence of support refers to this narrative set and is not a conclusion about all *Glomeromycota* research.

Theme	Finding in This Review	Claim Not Supported by This Study Set
Antimicrobial compounds	Rice resistance and sorghum metabolites [11,12]; tomato disease and receptor experiments [95,96,97].	No included study established production of a purified antimicrobial compound by *Glomeromycota* or tested it as a fungal-derived drug candidate.
Antioxidant activity and phenolics	Plant phenolic and antioxidant-related responses varied across Passiflora, Medicago, lettucem and aromatic plants [19,20,70,89,93].	The measured plant responses do not establish that the fungus directly synthesizes the phenolics or that a plant extract has clinical efficacy.
Antitumoral compounds	No tumor-cell or antitumor assay was identified among the 102 studies discussed.	Neither the presence of a specialized plant metabolite nor medicinal interest demonstrates antitumoral activity.
Enzymes	Host-lipid enzymes and bacterial phosphatase-related functions were investigated within symbiosis and the hyphosphere [3,4,5,30,44,45,46,61,62].	No included study demonstrated production, purification, or industrial performance of a *Glomeromycota* enzyme.
Organic acids	Plant metabolomes included organic-acid-related responses in sorghum, Anchusa, peanut, and strawberry [12,16,76,98].	These observations do not demonstrate fungal organic-acid production at a recoverable bioprocess scale.
Pigments	Plant alkannin/shikonin-related compounds [18] and grape anthocyanins [92] changed following inoculation.	This is modulation of plant compounds; fungal pigment biosynthesis, extraction, and yield were not demonstrated.
Conditions affecting bioactive outcomes	Responses depended on tissue, strain, genotype, soil, water, temperature, cultivation, or storage [2,11,12,16,17,18,19,20,67,70,89,90,91,92,93,94].	Effects cannot be generalized without specifying the host–isolate–environment combination and the measured chemical endpoint.
Other applications supported	Research also measured nutrient flux, soil hydraulics/carbon, defense, crop performance, and contaminant behavior [39,40,41,42,43,44,45,46,47,48,49,50,51,52,53,54,55,56,57,58,59,60,61,62,77,78,79,80,81,82,83,84,85,86,87,88,99,100,101,102].	These outcomes are biotechnologically relevant but should be described by their own endpoints rather than relabeled as fungal natural-product production.

**Table 4:** Biological basis and development constraints of AMF culture approaches. This is a qualitative comparison, not a measured productivity or cost ranking.

Culture Approach	Biological Basis and Evidence	Scale-Up and Comparison Limits
Host-associated propagation	Living host/root carbon supply; dependence supported by lipid experiments [3,4].	Manage host biomass, substrate, and contamination; viable output may include spores, roots, and hyphae.
Root–organ cultivation	Controlled living-root supply and experimental process optimization [37]; storage structures can develop in excised trap roots [38].	Aseptic root maintenance and downstream separation add process requirements; no harmonized economic ranking established.
Asymbiotic-defined medium cultivation	External fatty-acid supply supports growth and infective spores [5,21,31].	Strain/media dependence and post-storage infectivity require testing; no universal gain in cost or yield demonstrated.

## Data Availability

No new data were created or analyzed in this study. Data sharing is not applicable to this article.
